# Uric acid-to-albumin ratio as a cardiometabolic marker for predicting adverse outcomes in patients with atrial fibrillation: evidence from two independent cohorts

**DOI:** 10.3389/fendo.2026.1786997

**Published:** 2026-02-13

**Authors:** Aobo Gong, Ying Cao, Zexi Li, Fanghui Li, Wenjie Li, Bangjiaxin Ren, Xianjin Hu, Yifan Zhou, Rui Zeng

**Affiliations:** Department of Cardiology, West China Hospital, Sichuan University West China School of Medicine, Chengdu, China

**Keywords:** atrial fibrillation, cardiometabolic burden, external validation, metabolic disorders, risk stratification, uric acid-to-albumin ratio

## Abstract

**Introduction:**

Atrial fibrillation (AF) is closely associated with metabolic dysfunction. The uric acid–to–albumin ratio (UAR), integrating oxidative stress, inflammation, and nutritional status, reflect cardiometabolic burden, but evidence linking UAR to AF prognosis remains limited.

**Methods:**

We analyzed clinical data from 1,908 AF patients at West China Hospital, with external validation from the MIMIC database (n=1,366). Associations were assessed using Kaplan–Meier analyses, restricted cubic splines, and multivariable Cox proportional hazards models. Incremental prognostic value beyond the CHA_2_DS_2_-VASc score was evaluated in both cohorts. Exploratory machine learning and SHAP analyses were employed to assess the variable importance of UAR. Subgroup and sensitivity analyses were performed in primary cohort, including additional cardiometabolic adjustment, analyses with cardiac mortality, competing risk models, and longer follow-up.

**Results:**

Baseline characteristics differed across UAR quartiles, with high UARs associated with substantial burdens of metabolic comorbidities, heart failure, renal dysfunction, and elevated inflammatory and cardiac biomarkers. Mortality was higher in the highest UAR quartile (log-rank P<0.001). In the primary cohort, restricted cubic splines showed a J-shaped association between UAR and 1-year mortality (P for nonlinearity <0.001). In fully adjusted Cox models, UAR (per SD) predicted 1-year all-cause mortality in the primary cohort (HR 1.162, 95% CI 1.036–1.304) and in the MIMIC cohort (HR 1.137, 95% CI 1.092–1.185). Adding UAR to the CHA_2_DS_2_-VASc score improved discrimination (C-index 0.654 to 0.692; P = 0.001), reclassification (continuous NRI 0.178), calibration, and clinical net benefit, with consistent incremental performance in the MIMIC cohort. In both cohorts, SHAP analysis consistently identified UAR as one of the major contributors to mortality prediction. Findings were consistent across subgroups and sensitivity analyses.

**Conclusion:**

UAR is an independent predictor of mortality in AF and captures cardiometabolic remodeling beyond conventional risk assessment. As a readily available biomarker, UAR may facilitate metabolically guided risk stratification and individualized management in AF populations.

## 1 Introduction

Atrial fibrillation (AF) is the most common cardiac arrhythmia worldwide and associated with substantial morbidity and mortality (1). In addition to its established relationship to stroke and heart failure (HF), AF frequently occurs in patients with a high burden of cardiometabolic comorbidities, including metabolic dysfunction, systemic inflammation, and impaired nutritional status (1–4). These factors contribute to marked heterogeneity in clinical outcomes among patients with AF. However, commonly used AF risk stratification tools, such as the CHA_2_DS_2_-VASc score, primarily focus on demographic characteristics and comorbidities, and may not adequately capture differences in metabolic and inflammatory burden that are relevant to prognosis (5).

Serum uric acid (UA) and albumin (ALB) are commonly available metabolic biomarkers that reflect systemic inflammation and oxidative stress (4, 6–8). Elevated UA levels and reduced ALB concentrations are both associated with poor prognosis in patients with AF (9, 10). However, individual markers may insufficiently reflect the overall cardiometabolic burden and are susceptible to influence from comorbid conditions, such as impaired renal function (6, 11–13). Recently, the uric acid-to-albumin ratio (UAR), a composite index incorporating both UA and ALB, has been proposed and its clinical use has been validated in cardiometabolic conditions, such as coronary artery disease (CAD) (14) and diabetes mellitus (DM) (13). By jointly reflecting oxidative stress, inflammation, and nutritional status, UAR may provide a more comprehensive assessment of cardiometabolic risk than individual biomarkers (13). Patients with AF often exhibit different degrees of metabolic and inflammatory abnormalities, suggesting a potential role for UAR in risk stratification (3, 5). However, evidence regarding its prognostic value in patients with AF remains limited.

In this study, we aimed to test the hypothesis that the UAR, as a composite cardiometabolic marker, is independently associated with long-term mortality risk in patients with AF. Furthermore, we sought to evaluate whether UAR could provide incremental prognostic value beyond established risk stratification scores. By integrating a readily available composite biomarker that reflects metabolic, inflammatory, and nutritional status, this study may offer novel insights into risk assessment and help inform individualized management strategies for patients with AF.

## 2 Materials and methods

### 2.1 Study design and data sources

This retrospective study used two independent cohorts. The primary cohort was from West China Hospital, Chengdu, China, and included consecutive admissions between January and June 2020 with a discharge diagnosis of AF. The study protocol was approved by the Ethics Committee of West China Hospital, Sichuan University (Approval No. 2022-306).

External validation relied on the Medical Information Mart for Intensive Care (MIMIC) databases, including MIMIC-IV (version 3.1) and the CareVue subset of MIMIC-III, both from Beth Israel Deaconess Medical Center, Boston, Massachusetts, United States. In MIMIC, AF was identified using ICD-9/10 codes 427.31, I48, I48.0, I48.1, I48.2, I48.9, I48.11, I48.19, I48.20, and I48.21. The MIMIC databases are de-identified and publicly accessible after completion of required training and a data use agreement.

### 2.2 Study population

The same eligibility criteria were applied to both cohorts. Patients were included if they were aged ≥18 years and had AF documented for the hospitalization. For patients with multiple hospitalization records, only the data from their first hospitalization was collected. Patients with a hospital stay of less than 1 day or missing UA and ALB measurement were excluded.

### 2.3 Data extraction and covariates

West China Hospital data were extracted from the electronic medical record system. We collected age and sex, comorbidities (including HT, DM, stroke/transient ischemic attack [TIA], HF, and chronic obstructive pulmonary disease [COPD]), and medication information during hospitalization (oral anticoagulants [OAC], antiplatelet agents, β-blockers, statins, and other cardiovascular drugs). Laboratory variables included γ-glutamyltransferase (GGT), triglycerides, admission blood glucose, UA, ALB, high-density lipoprotein cholesterol (HDL-C), low-density lipoprotein cholesterol (LDL-C), lactate dehydrogenase (LDH), aspartate aminotransferase (AST), hemoglobin, white blood cell count, N-terminal pro-B-type natriuretic peptide (NT-proBNP), cardiac troponin T (cTnT), and other available indicators.

For MIMIC-IV and MIMIC-III CareVue, data were queried using PostgreSQL based on the official schemas. We extracted demographics, comorbidities, laboratory values, medication information, and mortality outcomes. Several biomarkers (notably GGT, LDL-C, HDL-C, NT-proBNP, and cTnT) showed substantial missingness in MIMIC; analyses that required those markers were therefore not performed in the validation cohort.

All laboratory measurements were taken as the earliest recorded values after hospital admission or ICU admission. Units were harmonized across datasets before calculations.

### 2.4 Exposure definition

The UAR was defined as serum UA (μmol/L) to serum ALB (g/L). Body mass index (BMI) was calculated as weight (kg)/height^2^ (m²). Estimated glomerular filtration rate (eGFR) was calculated from serum creatinine (mg/dL) using the 2009 CKD-EPI equation without a race coefficient.

### 2.5 Follow-up and outcomes

The primary outcome was 1-year all-cause mortality. In the West China Hospital cohort, vital status was ascertained through the hospital electronic medical record, outpatient visits, online follow-up, and structured telephone interviews. In the MIMIC databases, mortality information was obtained from in-hospital death records and Social Security follow-up data. For all analyses, time zero was defined as the date of hospital admission. The secondary outcome was cardiovascular death. Owing to the limitation of the MIMIC databases, analyses of cardiovascular mortality and longer follow-up were restricted to the West China Hospital cohort.

### 2.6 Statistical analysis

Baseline characteristics across UAR quartiles were compared using the Kruskal–Wallis test for continuous variables and chi-square or Fisher’s exact tests for categorical variables. Missing covariates were addressed using multiple imputation with chained equations (20 datasets per cohort), and estimates were combined according to Rubin’s rules (Details of missing data for both cohorts are provided in **Supplementary Table 1**). In the primary cohort, Kaplan–Meier curves with log-rank tests were used to assess 1-year mortality. Potential nonlinear relationships were explored using restricted cubic splines (RCS). Cox proportional hazards models were constructed to estimate hazard ratios (HR; per SD increase) and 95% confidence interval (CI), with sequential adjustment for demographics, comorbidities, medications, and available laboratory measurements (Detailed covariate lists are provided in **Supplementary Table 2**). Proportional hazards assumptions were examined and any violations were appropriately handled. External validation was performed in the MIMIC cohort, with UAR standardized to the primary cohort.

To evaluate the incremental prognostic contribution of UAR beyond the CHA_2_DS_2_-VASc score, models with and without UAR were compared using Harrell’s C-index, time-dependent area under the curve (AUC), calibration, decision curve analysis, and net reclassification improvement (NRI)/integrated discrimination improvement (IDI) in both cohorts. Exploratory machine learning models (random survival forests, extreme gradient boosting, and gradient boosting machines) were additionally used solely to investigate potential nonlinear associations and to gain insight into the relative importance of UAR among clinical variables using Shapley additive explanations (SHAP). Prespecified subgroup and sensitivity analyses were conducted in the primary cohort, including additional adjustment for advanced biomarkers in Cox proportional hazards models, cardiovascular mortality, competing risk analyses using Fine–Gray models, and 4-year outcomes. All analyses were carried out in R (version 4.5.1). A two-sided P value <0.05 was considered statistically significant. Further statistical details are provided in the **Supplementary Material 2**.

## 3 Results

### 3.1 Study population and baseline characteristics

**Figure 1:**
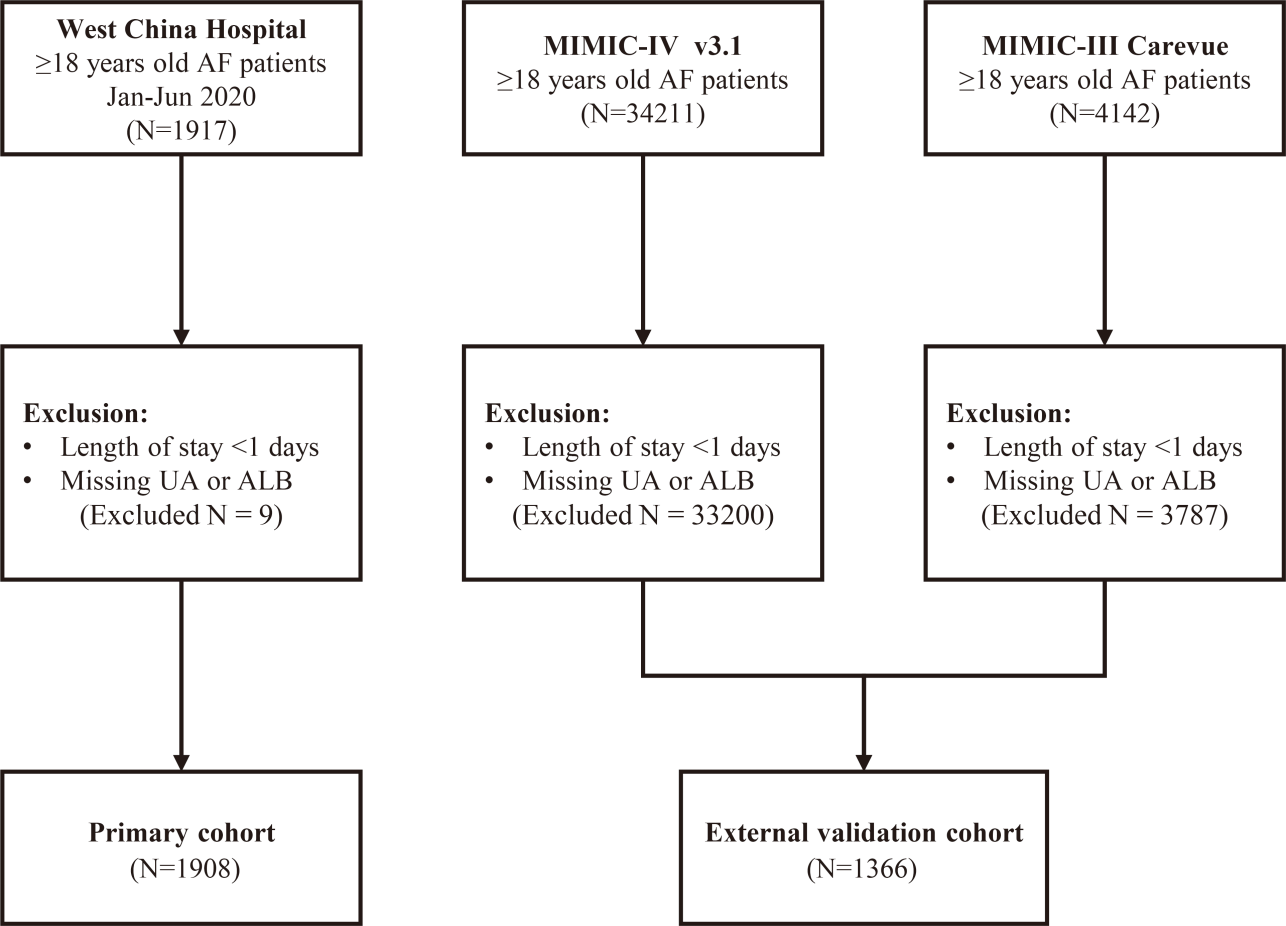
Flow chart. AF, atrial fibrillation; MIMIC, Medical Information Mart for Intensive Care; UA, serum uric acid; ALB, serum albumin.

After applying the inclusion and exclusion criteria, 1908 patients were included in the primary cohort (**Figure 1**). The mean age was 67.9 years and 53.8% of the participants were male. The external validation cohort from the MIMIC database comprised 1366 patients, with an average age of 71.8 years and 60.2% males.

In the primary cohort, baseline characteristics significantly differed across UAR quartiles (**Table 1**). Higher UAR levels were associated with a greater proportion of males and higher CHA_2_DS_2_-VASc scores. CAD, DM, and COPD were also more common among patients with elevated UAR. The prevalence of HF markedly increased from 33.1% in the lowest to 70.2% in the highest UAR group (P<0.001). eGFR progressively declined with increasing UAR, accompanied by higher levels of NT-proBNP and cTnT. Markers of inflammation and oxidative stress, including white blood cell count, AST, GGT, and LDH, showed graded increases, whereas HDL-C decreased across quartiles. Moreover, OAC use was less frequent in the highest UAR quartile, while other cardiovascular therapies were largely comparable across groups.

**Table 1:** Baseline characteristics for primary cohort.

	T1(n=477)	T2(n=477)	T3(n=477)	T4(n=477)	*P*.value
	UAR<6.890	UAR:6.890-8.728	UAR:8.728-11.142	UAR>11.142	
Male	182 (38.2%)	234 (49.1%)	298 (62.5%)	312 (65.4%)	<0.001
Age	70.0 [61.0;78.0]	67.0 [59.0;76.0]	67.0 [56.0;77.0]	72.0 [61.0;80.0]	<0.001
Weight (kg)	59.0 [50.0;66.0]	62.0 [55.0;70.0]	65.0 [56.0;74.0]	62.5 [53.9;71.0]	<0.001
Height (m)	1.60 [1.55;1.67]	1.60 [1.55;1.68]	1.63 [1.56;1.70]	1.63 [1.57;1.70]	<0.001
CHA2DS2-VASc	3.00 [2.00;5.00]	3.00 [2.00;4.00]	3.00 [2.00;4.00]	4.00 [2.00;5.00]	<0.001
Comorbidity (%)					
DM	102 (21.4%)	84 (17.6%)	96 (20.1%)	120 (25.2%)	0.037
HT	216 (45.3%)	237 (49.7%)	211 (44.2%)	240 (50.3%)	0.145
HF	158 (33.1%)	181 (37.9%)	261 (54.7%)	335 (70.2%)	<0.001
Stroke/TIA	113 (23.7%)	77 (16.1%)	62 (13.0%)	87 (18.2%)	<0.001
SE	10 (2.10%)	12 (2.52%)	6 (1.26%)	18 (3.77%)	0.083
CAD	96 (20.1%)	109 (22.9%)	122 (25.6%)	151 (31.7%)	<0.001
PAD	36 (7.55%)	39 (8.18%)	38 (7.97%)	39 (8.18%)	0.982
COPD	60 (12.6%)	46 (9.64%)	44 (9.22%)	76 (15.9%)	0.004
OSAS	4 (0.84%)	2 (0.42%)	2 (0.42%)	4 (0.84%)	0.778
Hyperthyroidism	10 (2.10%)	6 (1.26%)	3 (0.63%)	9 (1.89%)	0.226
Hypothyroidism	14 (2.94%)	15 (3.14%)	20 (4.19%)	21 (4.40%)	0.533
Malignant tumor	38 (7.97%)	36 (7.55%)	32 (6.71%)	29 (6.08%)	0.670
Laboratory tests					
RBC (k/ul)	4.10 [3.66;4.53]	4.36 [3.92;4.85]	4.35 [3.83;4.85]	4.05 [3.43;4.70]	<0.001
HB (g/dl)	12.6 [11.1;13.8]	13.3 [11.8;14.7]	13.4 [11.7;14.7]	12.3 [10.2;14.1]	<0.001
WBC (k/ul)	6.21 [4.75;8.53]	6.01 [4.95;7.69]	6.32 [5.04;7.95]	7.07 [5.43;9.18]	<0.001
PLT (k/ul)	161 [119;207]	159 [120;195]	156 [117;197]	146 [110;192]	0.015
Tbil(mg/dl)	0.81 [0.57;1.11]	0.80 [0.58;1.03]	0.81 [0.61;1.13]	0.85 [0.60;1.33]	0.039
ALT (IU/L)	18.0 [13.0;27.0]	19.0 [13.0;27.0]	20.0 [14.0;32.0]	21.0 [13.0;38.0]	0.005
AST (IU/L)	23.0 [18.0;31.0]	22.0 [18.0;29.0]	24.0 [19.0;31.0]	27.0 [21.0;43.0]	<0.001
GGT (IU/L)	28.0 [17.0;52.0]	29.0 [18.0;52.0]	40.0 [21.0;66.0]	49.0 [27.0;95.0]	<0.001
eGFR (mL/min/1.73 m²)	84.4 [73.2;94.1]	78.9 [66.1;91.2]	73.8 [58.7;89.2]	53.4 [36.2;72.4]	<0.001
CK (IU/L)	72.0 [48.0;106]	70.0 [50.0;103]	76.0 [52.0;116]	71.0 [46.0;120]	0.160
LDH (IU/L)	193 [162;238]	186 [163;222]	194 [167;245]	231 [183;320]	<0.001
FIB (mg/dl)	271 [228;353]	266 [223;319]	275 [231;334]	285 [226;361]	0.020
AGLU (mg/dl)	108 [91.8;155]	102 [89.6;135]	105 [90.0;144]	122 [94.9;165]	<0.001
LDL-C (mg/dl)	81.2 [58.9;106]	83.5 [61.9;102]	82.0 [60.3;107]	70.0 [50.7;92.0]	<0.001
TG (mg/dl)	95.7 [71.7;132]	103 [77.9;143]	105 [75.3;156]	103 [77.1;144]	0.005
HDL-C (mg/dl)	47.2 [36.7;56.9]	44.5 [37.1;53.0]	41.4 [33.3;49.9]	36.3 [28.2;44.5]	<0.001
NT-proBNP (pg/ml)	755 [300;1635]	901 [330;1898]	1238 [552;2564]	3220 [1441;9073]	<0.001
cTnT(ng/ml)	0.01 [0.01;0.03]	0.01 [0.01;0.03]	0.02 [0.01;0.03]	0.04 [0.02;0.08]	<0.001
Medication use (%)					
OAC	304 (63.7%)	329 (69.0%)	329 (69.0%)	273 (57.2%)	<0.001
Antiplatelet drugs	59 (12.4%)	85 (17.8%)	81 (17.0%)	80 (16.8%)	0.093
ANRI/ACEI/ARB	101 (21.2%)	111 (23.3%)	104 (21.8%)	80 (16.8%)	0.078
β blocker:	147 (30.8%)	145 (30.4%)	156 (32.7%)	138 (28.9%)	0.654
Statin:	139 (29.1%)	139 (29.1%)	147 (30.8%)	135 (28.3%)	0.857
SGLT2:	4 (0.84%)	5 (1.05%)	2 (0.42%)	11 (2.31%)	0.041

UAR, uric acid-to-albumin ratio; DM, diabetes mellitus; HT, hypertension; HF, heart failure; TIA, transient ischemic attack; SE, systemic embolism; CAD, coronary artery disease; PAD, peripheral arterial disease; COPD, chronic obstructive pulmonary disease; OSAS, obstructive sleep apnea syndrome; RBC, red blood cell count; HB, hemoglobin; WBC, white blood cell count; PLT, platelet count; Tbil, total bilirubin; ALT, alanine aminotransferase; AST, aspartate aminotransferase; GGT, γ-glutamyltransferase; eGFR, estimate glomerular filtration rate; CK, creatine kinase; LDH, lactate dehydrogenase; FIB, fibrinogen; AGLU, admission blood glucose; LDL-C, low-density lipoprotein cholesterol TG, triglyceride; HDL-C, high-density lipoprotein cholesterol; NT-proBNP, N-terminal pro-B-type natriuretic peptide; cTnT, troponin T; OAC, oral anticoagulant; ARNI, angiotensin receptor neprilysin inhibitor; ACEI, angiotensin converting enzyme inhibitors; ARB, angiotensin receptor blocker; SGLT2, sodium-dependent glucose transporters 2.

Generally similar trends were observed in the validation cohort (**Supplementary Table 3**). Higher UAR was consistently associated with older age, higher CHA_2_DS_2_-VASc scores, a greater burden of HF and metabolic comorbidities, and worse renal function. However, in contrast to the primary cohort, OAC and antiplatelet drugs were more commonly used in individuals with higher UAR levels.

### 3.2 Association of UAR with 1-year all-cause mortality

**Figure 2:**
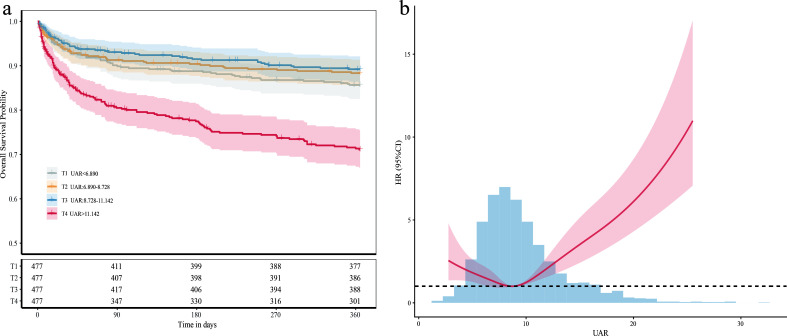
**(a)** Kaplan–Meier curves for 1-year all-cause mortality according to quartiles of the UAR. **(b)** Nonlinear association between the UAR and 1-year all-cause mortality assessed using restricted cubic splines. UAR, uric acid-to-albumin ratio; HR, hazard ratio; CI, confidence interval.

Kaplan–Meier survival curves for 1-year all-cause mortality are shown in **Figure 2a**. There were no significant differences in survival among the first three quartiles (log-rank P >0.05). In contrast, patients in the highest quartile showed a lower survival probability (log-rank P<0.001). RCS analysis was conducted to assess the continuous association between UAR and 1-year all-cause mortality (**Figure 2b**). A five-knot model achieved the lowest Akaike information criterion (5th, 35th, 50th, 65th, and 95th percentiles). A J-shaped association was observed between UAR and patient mortality risk (P for nonlinearity <0.001). The mortality risk was lowest when UAR was close to the median range. Above this threshold, further increases in UAR were associated with a continuous upward trend in mortality risk.

In Cox proportional hazards analyses, UAR was associated with 1-year all-cause mortality across four models (**Table 2**). In Cox proportional hazards analyses, a high UAR was associated with an increased risk of 1-year mortality. Effect estimates decreased but were statistically significant after accounting for a broad range of demographic, clinical, renal, metabolic, and laboratory covariates. In the fully adjusted model, each 1-SD increase in UAR was associated with a 16.2% higher risk of mortality (HR 1.162, 95% CI 1.036–1.304; P = 0.011).

**Table 2:** Association between UAR and 1-year all-cause mortality in Cox proportional hazards analyses for primary cohort.

Model	Demographics	BMI	Comorbidities	Medications	Labs	UAR (per 1-SD)* HR (95%CI)	P.value
Model1						1.541(1.412-1.680)	<0.001
Model2	**✓**					1.443(1.323-1.574)	<0.001
Model3	**✓**	**✓**	**✓**	**✓**		1.375(1.251-1.511)	<0.001
Model4	**✓**	**✓**	**✓**	**✓**	**✓**	1.162(1.036-1.304)	0.011

*UAR modeled per 1-SD increase. Detailed covariate lists are provided in **Supplementary Table S2**.

UAR, uric acid-to-albumin ratio; BMI, body mass index; SD, standard deviation; HR, hazard ratio; CI, confidence interval.

In the MIMIC cohorts, higher UAR was consistently associated with an increased risk of 1-year all-cause mortality in Cox analyses (**Table 3**). The association remained significant after adjustment for demographic characteristics, comorbid conditions, clinical variables, renal function, and laboratory measures available in the external dataset. In the fully adjusted MIMIC model, UAR continued to show a significant association with 1-year mortality (HR 1.137, 95% CI 1.092–1.185; P<0.001).

**Table 3:** Association between UAR and 1-year all-cause mortality in Cox proportional hazards analyses for external validation cohort.

Model	Demographics	BMI	Comorbidities	Medications	Labs	UAR (per 1-SD)* HR (95%CI)	P.value
Model1						1.098(1.061-1.137)	<0.001
Model2	**✓**					1.102(1.064-1.143)	<0.001
Model3	**✓**	**✓**	**✓**	**✓**		1.120(1.081-1.161)	<0.001
Model4	**✓**	**✓**	**✓**	**✓**	**✓**	1.137(1.092-1.185)	<0.001

*UAR was scaled using the SD derived from the primary cohort. Detailed covariate lists are provided in **Supplementary Table S2**.

UAR, uric acid-to-albumin ratio; BMI, body mass index; SD, standard deviation; HR, hazard ratio; CI, confidence interval.

### 3.3 Incremental prognostic value beyond CHA_2_DS_2_-VASc

**Figure 3:**
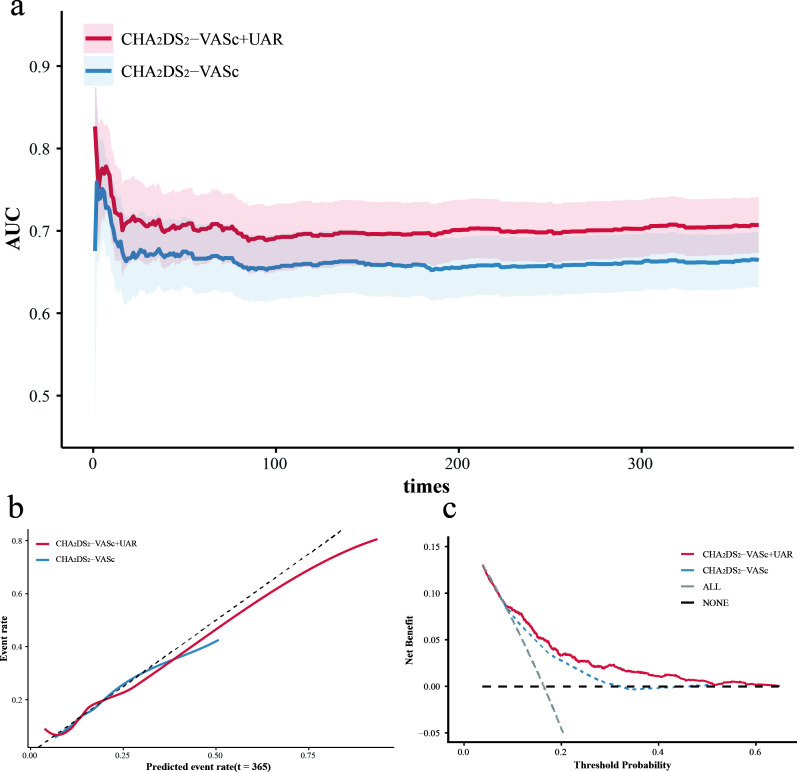
**(a)** Time-dependent AUC comparison of CHA_2_DS_2_-VASc with and without UAR in primary cohort. **(b)** Calibration curves comparing CHA_2_DS_2_-VASc models with and without UAR in primary cohort. **(c)** Decision curve analysis of CHA_2_DS_2_-VASc models with and without UAR in primary cohort. AUC, area under curve; UAR, uric acid-to-albumin ratio.

In the primary cohort, the addition of UAR to the CHA_2_DS_2_-VASc score improved the prediction of 1-year mortality risk. The combined model demonstrated higher time-dependent AUCs throughout follow-up (**Figure 3a**), with statistically significant differences at 30 days (P = 0.046) and 360 days (P = 0.029). Increases were observed at 90 and 180 days, but these did not reach statistical significance (P = 0.070 and 0.052). The C-index increased from 0.654 for the CHA_2_DS_2_-VASc score to 0.692 for the combined model (P = 0.001). Calibration curves (**Figure 3b**) indicated that the predicted probabilities of 1-year mortality from both models closely approximated the observed probabilities. The delta Brier score was 0.006 (95% CI 0.003–0.009). Decision curve analysis (**Figure 3c**) suggested a greater net benefit across the full range of threshold probabilities for the combined model. Reclassification analyses yielded an IDI of 0.047 (95% CI 0.025–0.070) and a continuous NRI of 0.178 (95% CI 0.110–0.252).

For external validation, the baseline risk was estimated using the same coefficients derived from the primary cohort. The time-dependent AUC increased throughout follow-up (**Supplementary Figure 1a**), with P values of 0.001, 0.007, 0.008, and 0.039 at 30, 90, 180, and 360 days, respectively. The C-index increased from 0.516 to 0.546 (P = 0.003). Calibration analysis (**Supplementary Figure 1b**) indicated an underestimation of absolute risk in the lower predicted range in the external cohort for both models. Compared with the conventional model, the model incorporating UAR showed better agreement with observed outcomes in the intermediate to higher risk range. Consistently, the delta Brier score suggested a reduction in overall prediction error (ΔBrier = 0.045, 95% CI 0.035–0.055). Reclassification analyses showed a continuous NRI of 0.125 (95% CI 0.018–0.225) and an IDI of 0.041 (95% CI 0.025–0.059).

### 3.4 Machine learning analyses

Machine learning analyses were conducted as exploratory assessments of variable importance and potential nonlinear relationships. Model development and selection were performed using one imputed dataset from the primary cohort. Among the evaluated algorithms, random survival forests demonstrated the highest C-index. Therefore, it was used for subsequent analyses.

The final random survival forests model was applied to all imputed datasets in the primary cohort and the external validation cohort. In the external cohort, model discrimination was moderate, with a mean C-index of 0.670 (SD 0.002). AUCs in the external cohort were 0.731 at 30 days, 0.716 at 90 days, 0.719 at 180 days, and 0.707 at 365 days.

**Figure 4:**
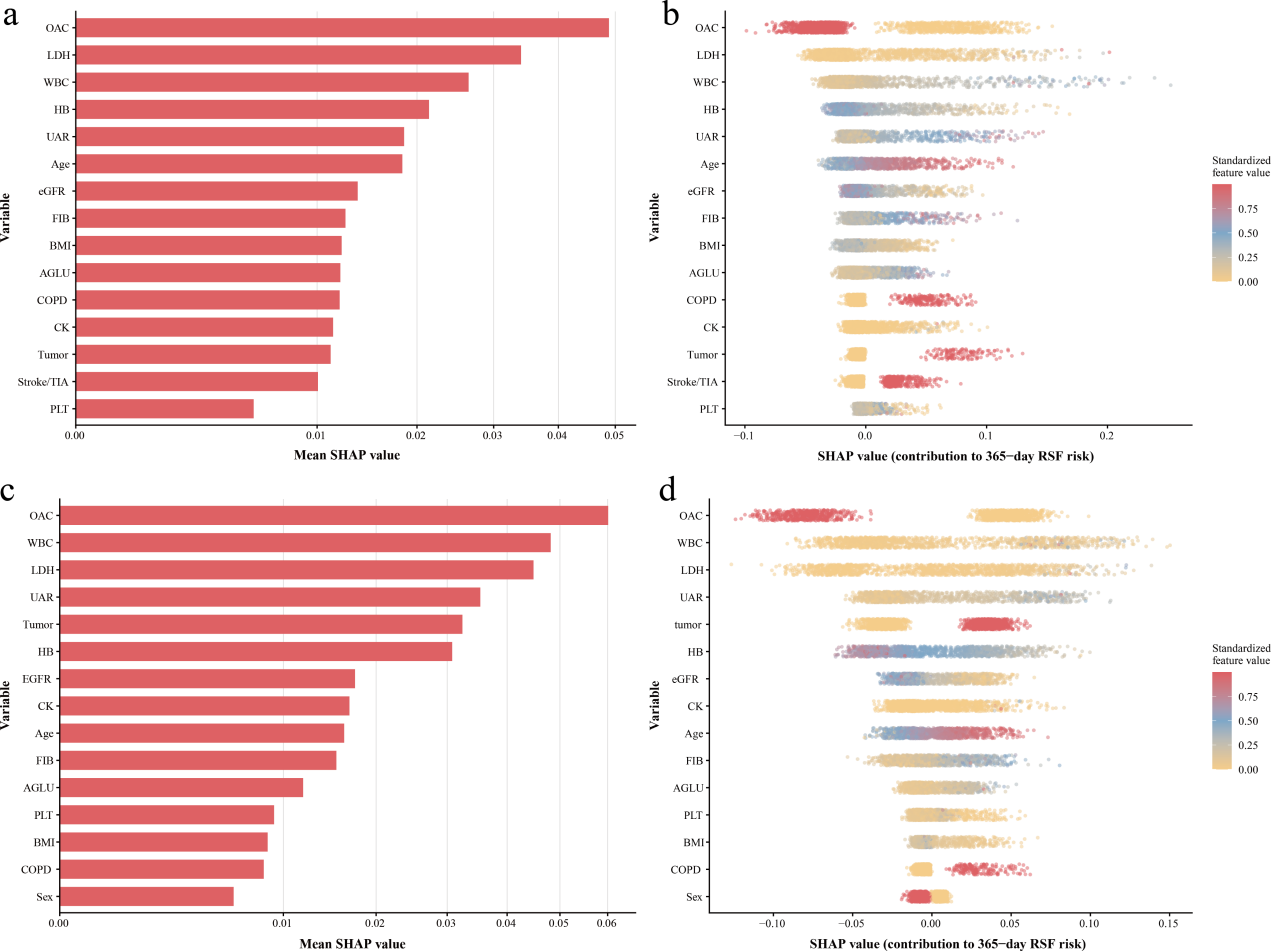
**(a)** RSF-SHAP variable importance in primary cohort. **(b)** RSF-SHAP beeswarm plot showing feature effects in primary cohort. **(c)** RSF-SHAP variable importance in external validation cohort. **(d)** RSF-SHAP beeswarm plot showing feature effects in external validation cohort. RSF, random survival forests; SHAP, Shapley additive explanations; OAC, oral anticoagulant; LDH, lactate dehydrogenase; WBC, white blood cell count; HB, hemoglobin; UAR, uric acid-to-albumin ratio; eGFR, estimate glomerular filtration rate; FIB, fibrinogen; BMI, body mass index; AGLU, admission blood glucose; COPD, chronic obstructive pulmonary disease; CK, creatine kinase; TIA, transient ischemic attack; PLT, platelet count.

SHAP values were calculated for 365-day mortality using one imputed dataset. UAR was one of the most influential predictors, alongside several key clinical and laboratory variables (**Figure 4a**). Stability analyses performed using three additional imputed datasets yielded similar rankings, with UAR consistently appearing among the top contributing variables (**Supplementary Figure 2**). The beeswarm plot showed that high UAR values produced high SHAP values, with a stable distribution across the range (**Figure 4b**). To further evaluate robustness, the final random survival forest model was applied to the external cohort (**Figures 4c, d**). UAR maintained a high level of variable importance. In robustness analyses across three additional imputed external datasets, the UAR was consistently ranked the fourth most influential variable (**Supplementary Figure 3**).

### 3.5 Subgroup and sensitivity analyses

**Figure 5:**
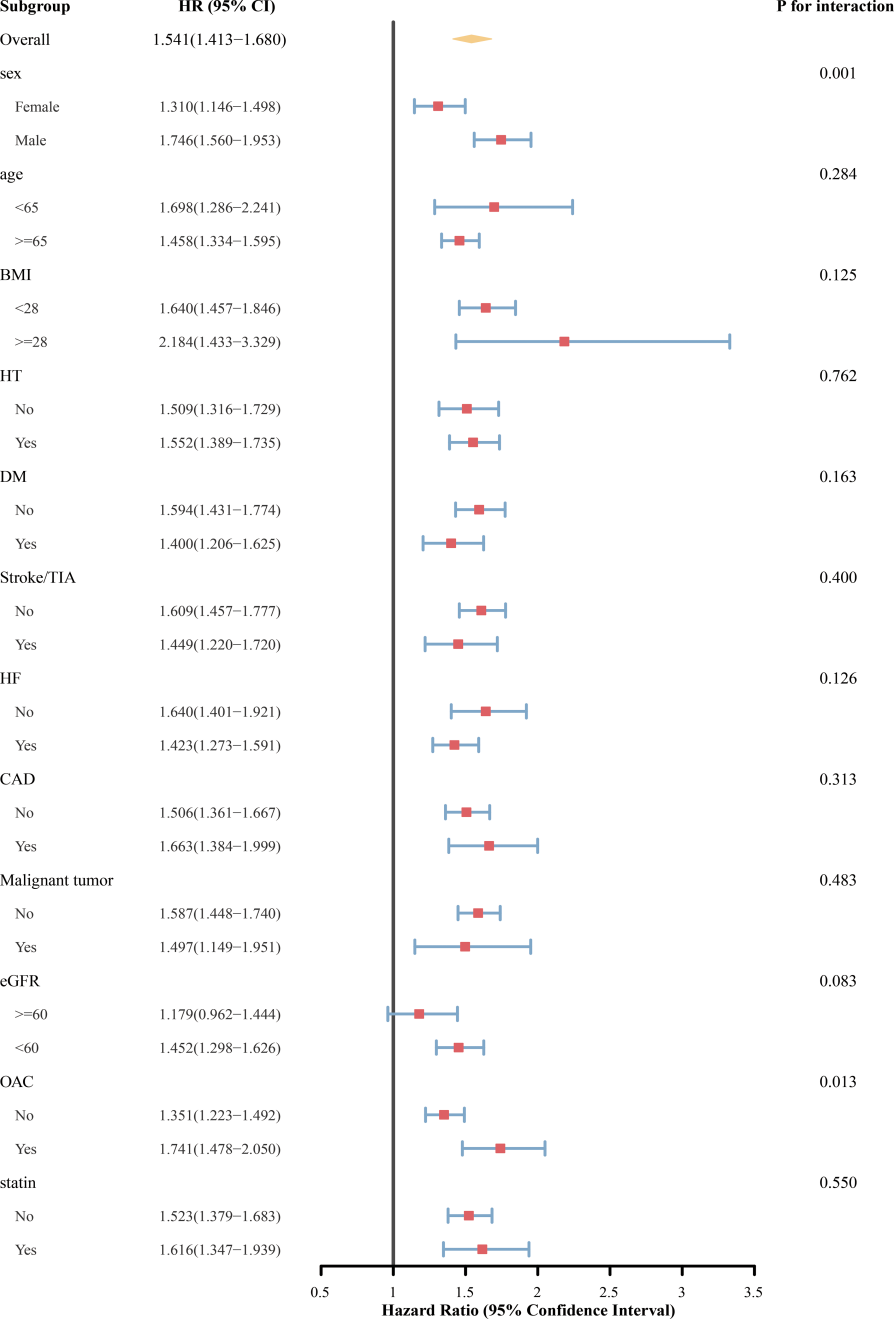
Subgroup analyses of the association between UAR and 1-year all-cause mortality in primary cohort. UAR, uric acid-to-albumin ratio; HR, hazard ratio; CI, confidence interval; BMI, body mass index; HT, hypertension; DM, diabetes mellitus; TIA, transient ischemic attack; HF, heart failure; CAD, coronary artery disease; eGFR, estimate glomerular filtration rate; OAC, oral anticoagulant.

In the primary cohort, high UAR was associated with increased 1-year all-cause mortality across most subgroups (**Figure 5**). The association was consistent across subgroups of age, BMI, HT, DM, stroke/TIA, HF, CAD, and statin use, with no significant interactions observed (all P for interaction >0.05). A significant interaction was observed by sex (P for interaction = 0.001), with a stronger association between the UAR and mortality in men than that in women. OAC use also influenced the association (P for interaction = 0.013), with higher mortality risk among patients receiving OAC. The interaction with renal function was marginally significant (P for interaction = 0.083). UAR was significantly associated with mortality in patients with eGFR <60 mL/min/1.73 m², but the association was not statistically significant in those with preserved renal function.

**Figure 6:**
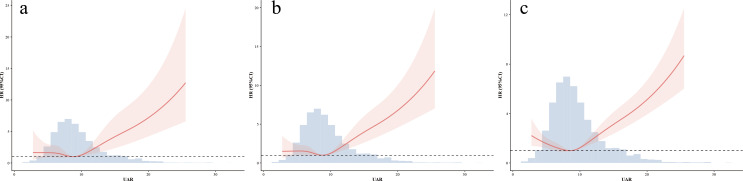
**(a)** Nonlinear association between the UAR and 1-year cardiac mortality assessed using restricted cubic splines. **(b)** Nonlinear association between the UAR and 4-year cardiac mortality assessed using restricted cubic splines. **(c)** Nonlinear association between the UAR and 4-year all-cause mortality assessed using restricted cubic splines. UAR, uric acid-to-albumin ratio; HR, hazard ratio; CI, confidence interval.

After additional adjustment for GGT, LDL-C, triglyceride, HDL-C, NT-proBNP, and cTnT, the association between UAR and 1-year all-cause mortality remained significant (HR 1.130, 95% CI 1.006–1.269; P = 0.039, **Supplementary Table 4** presents the results of the Cox regression for full adjusted variables). For 1-year cardiovascular mortality, the UAR was associated with increased risk in the unadjusted Cox model (HR 1.13, 95% CI 1.10–1.17; P<0.001). This association remained significant after adjustment for the CHA_2_DS_2_-VASc score (HR 1.12, 95% CI 1.08–1.15; P<0.001). RCS analysis with five knots demonstrated a J-shaped association between UAR and 1-year cardiovascular mortality (P for non-linearity=0.020, **Figure 6a**). In competing risk analyses treating non-cardiovascular death as a competing event, cumulative incidence curves (**Supplementary Figure 4**) showed higher cardiovascular mortality in the highest UAR quartile compared with that in the lower quartiles (all adjusted P<0.001), whereas no significant differences were observed among the lower three quartiles. In the Fine–Gray sub-distribution hazard model adjusted for the CHA_2_DS_2_-VASc score, UAR remained independently associated with cardiovascular mortality (sub-distribution HR 1.10, 95% CI 1.06–1.14; P<0.001).

For long-term outcomes, a high UAR was consistently associated with increased 4-year all-cause and cardiovascular mortality. In unadjusted analyses, UAR was associated with both all-cause mortality (HR 1.11, 95% CI 1.09–1.13; P<0.001) and cardiovascular mortality (HR 1.13, 95% CI 1.10–1.16; P<0.001). These associations remained significant after adjustment for the CHA_2_DS_2_-VASc score (all-cause mortality: HR 1.10, 95% CI 1.08–1.11; cardiovascular mortality: HR 1.11, 95% CI 1.09–1.14; both P<0.001). RCS analyses demonstrated J-shaped relationships between UAR, and both 4-year cardiovascular mortality and all-cause (both P for non-linearity <0.001, **Figures 6b, c**).

## 4 Discussion

In this retrospective analysis of two independent cohorts, we observed a consistent association between UAR and both short- and long-term mortality among patients with AF. Higher UAR levels were associated with increased 1-year all-cause mortality, and this association persisted after adjustment for demographic characteristics, comorbidities, renal function, and metabolic parameters. RCS analyses suggested a J-shaped relationship, with the lowest risk observed at intermediate UAR levels. In addition, UAR showed incremental prognostic value beyond the CHA_2_DS_2_-VASc score in terms of discrimination and reclassification. Similar associations were observed in an external cohort from a different healthcare system, despite substantial differences in patient characteristics and clinical settings.

AF is regarded as a systemic condition with significant metabolic and inflammatory involvement, rather than a simple electrophysiological disorder (3, 15). Chronic inflammation, oxidative stress, neurohormonal activation, and metabolic imbalance jointly contribute to atrial structural and electrophysiological remodeling, ultimately leading to atrial cardiomyopathy (3). However, our results suggest that commonly used prognostic tools in AF may insufficiently reflect the individual variation in metabolic and inflammatory burden. Several cardiometabolic indices have been proposed to address this issue, such as the triglyceride-glucose and stress hyperglycemia index (16, 17). However, both indices rely on fasting glucose or glycated hemoglobin tests, which are not commonly prioritized for assessment in general AF population, thereby limiting their clinical applicability.

UAR combines two widely available laboratory parameters that are commonly measured in clinical practice. Elevated UA increases reactive oxygen species, activate the NLRP3 inflammasome, and enhance Kv1.5 expression (18–20). These changes impair mitochondrial function, shorten action potential duration, and disturb cardiomyocyte metabolism, contributing to AF (21). In addition, UA stimulates the TGF-β1/Smad2/3 pathway and promotes MMP-9 production, leading to atrial fibrotic remodeling (22, 23). Serum ALB acts as a major anti-inflammatory and antioxidant protein in the circulation (24). Its highly reactive Cys34 residue provides strong scavenging of reactive oxygen species, limiting oxidative stress and metabolic imbalance (25). ALB also modulates coagulation and platelet activity and carries drugs and hormones. Reduced ALB is indicative of increased oxidative stress, inflammatory activity, and metabolic burden, potentially impairing cardiac electrophysiology, facilitating AF, and correlating with poorer outcomes (26–28).

Our results support the concept that by integrating UA and ALB, UAR may serve as a composite surrogate capturing systemic metabolic and inflammatory status relevant to prognosis in AF, without requiring additional testing. Chen et al. found that the predictive value of UAR for poor prognosis in patients with DM is better than that of UA and ALB alone (13). Previous studies have demonstrated that elevated UAR is associated with adverse outcomes in HF, and other cardiovascular conditions (14, 29), and has also been linked to AF recurrence after catheter ablation (30). Moreover, given the high prevalence of frailty and malnutrition in patients with AF (31–33), UAR may indirectly reflect nutritional reserve and the capacity to tolerate physiological stress. In addition, UAR may reflect insulin resistance and broader cardiometabolic remodeling (21, 28, 34). These processes may promote atrial remodeling, endothelial dysfunction, renal impairment, and a higher HF burden in AF, which may help explain the increased mortality risk.

Our study identified a J-shaped association between the UAR and adverse outcomes in patients with AF. Elevated UAR values reflect a state of systemic inflammation and metabolic imbalance, potentially linked to frailty and malnutrition. In contrast, the very low UAR value may reflect a weakened antioxidant stress caused by low UA (6, 20), and the influence of unmeasured comorbidities and drugs, but its potential mechanism needs further investigation. Additionally, a large general population study using NHANES data also reported a J-shaped association between UAR and all-cause mortality and cardiovascular mortality, supporting our findings (35).

The incremental prognostic value over established risk scores suggests that UAR reflects pathophysiological burden not fully captured by routine clinical risk stratification tools. Moreover, the association between UAR and adverse outcomes was generally consistent across major clinical subgroups, although some interaction effects were observed. Differences according to sex and OAC use may reflect variation in baseline risk or treatment strategy (36). Our sensitivity analysis suggested that the association between UAR and all-cause mortality remained significant after further adjustment for biomarkers reflecting cardiac volume load and myocardial injury as well as lipid metabolic parameters, and UAR also showed consistent prognostic value for long-term (4-year) all-cause and cardiovascular mortality. Competing-risk analyses using Fine–Gray models further showed that accounting for non-cardiovascular death did not significantly change the association between UAR and cardiovascular mortality, supporting the robustness of UAR for risk stratification in AF. In clinical practice, UAR measurement is convenient, does not require fasting, and exhibits high reproducibility. These features make it suitable for repeated testing and for longitudinal risk stratification in patients with AF.

In the external validation, UAR remained independently associated with mortality and led to improved model discrimination and reclassification. Although model calibration was less optimal, the model tended to systematically underestimate the observed event rates in the low-risk stratum, whereas the inclusion of UAR was associated with a modest improvement in calibration across the intermediate to higher risk range. The observed improvement in the delta Brier score indicates better overall predictive performance. The calibration deviation is likely attributable to marked differences in baseline risk between general ward patients and critically ill populations, which limits the transferability of absolute risk estimates. However, the preserved discrimination and consistent associations across cohorts suggest that UAR reflects a potential cardiometabolic burden associated with long-term mortality in patients with AF across different clinical settings. In exploratory machine learning analyses focusing on variable importance, UAR was consistently ranked among the most influential variables, further supporting its potential relevance as a prognostic biomarker for the AF population.

To our knowledge, this study is the first to evaluate the prognostic value of UAR for adverse outcomes in patients with AF, and we applied multiple adjustment and sensitivity analyses, with external validation in an independent cohort, to assess the robustness of our findings. However, this study has some limitations. First, as a retrospective observational study, this study cannot establish a causal association between UAR and adverse outcomes in patients with AF, and our results do not support deriving a UAR cut-off for direct clinical use. Potential reverse causation is also possible; for example, severe illness may lower ALB and raise UA, which could increase UAR. Second, the missing data in the MIMIC cohort limited adjustment for certain laboratory parameters and precluded further analyses. In addition, the external validation cohort mainly included critically ill patients, which may limit generalizability to lower-risk individuals and contribute to suboptimal calibration. Third, UAR was measured only at admission, so we could not evaluate longitudinal UAR trajectories or their links to outcomes.

Future studies should prioritize prospective, multicenter cohorts with repeated UAR measurements to better establish temporality and reduce concerns about reverse causation. Causal inference could be further evaluated using Mendelian randomization with standard sensitivity analyses and transparent reporting (37). Additionally, causal mediation analysis, such as structural equation modeling could further quantitatively evaluate the potential mediating effects of UAR on adverse outcomes in AF patients through renal dysfunction, HF severity, and inflammatory burden, while simultaneously estimating direct effects (38). Finally, future work should derive UAR candidate thresholds using prespecified survival cutpoint methods (e.g., maximally selected rank statistics and changepoint approaches) (39), and validate them across different care settings and independent datasets beyond the MIMIC database to assess threshold stability and transportability for clinical risk stratification.

## 5 Conclusion

In patients with AF, the UAR was independently associated with short- and long-term mortality and provided additional cardiometabolic prognostic information beyond the CHA_2_DS_2_-VASc score. These associations were consistent across two independent cohorts from different clinical settings. As a readily available and inexpensive index, UAR could help clinicians recognize patients with a potential cardiometabolic and nutritional burden. Further prospective or longitudinal studies are needed to determine whether UAR-guided risk stratification strategies can improve clinical decision-making and patient outcomes.

## Data Availability

The datasets used and/or analyzed during the current study are available from the corresponding author on reasonable request.
