# Supplementary material for: Uric acid-to-albumin ratio as a cardiometabolic marker for predicting adverse outcomes in patients with atrial fibrillation: evidence from two independent cohorts
Source: Front Endocrinol (Lausanne). 2026 Feb 13;17:1786997. doi: 10.3389/fendo.2026.1786997 (PMC12945761; doi:10.3389/fendo.2026.1786997)
Supplement: Supplementary file 1 [file DataSheet1.docx]

Supplementary Material 1

**Supplementary Table 1**: Details of missing data for both cohorts.

|  | **Primary cohort** | **Validation cohort** |
| --- | --- | --- |
|  | **N=1908** | **N=1366** |
| Male | 0 | 0 |
| Age | 0 | 0 |
| Weight (kg) | 189 | 342 |
| Height (m) | 100 | 579 |
| **Comorbidity (%)** |  |  |
| DM | 0 | 0 |
| HT | 0 | 0 |
| HF | 0 | 0 |
| Stroke/TIA | 0 | 0 |
| SE | 0 | 0 |
| CAD | 0 | 0 |
| PAD | 0 | 0 |
| COPD | 0 | 0 |
| OSAS | 0 | 0 |
| Hyperthyroidism | 0 | 0 |
| Hypothyroidism | 0 | 0 |
| Malignant tumor | 0 | 0 |
| **Laboratory tests** |  |  |
| RBC (k/ul) | 0 | 1 |
| HB (g/dl) | 0 | 1 |
| WBC (k/ul) | 0 | 1 |
| PLT (k/ul) | 0 | 1 |
| Tbil(mg/dl) | 1 | 61 |
| ALT (IU/L) | 1 | 65 |
| AST (IU/L) | 1 | 59 |
| eGFR  (mL/min/1.73 m²) | 0 | 1 |
| CK (IU/L) | 5 | 541 |
| LDH (IU/L) | 5 | 137 |
| FIB (mg/dl) | 2 | 560 |
| AGLU (mg/dl) | 0 | 1 |
| GGT (IU/L) | 0 | - |
| LDL-C (mg/dl) | 0 | - |
| TG (mg/dl) | 0 | - |
| HDL-C (mg/dl) | 1 | - |
| NT-proBNP (pg/ml) | 169 | - |
| cTnT(ng/ml) | 321 | - |

DM: diabetes mellitus; HT: hypertension; HF: heart failure; TIA: transient ischemic attack; SE: systemic embolism; CAD: coronary artery disease; PAD: peripheral arterial disease; COPD: chronic obstructive pulmonary disease; OSAS: obstructive sleep apnea syndrome; RBC: red blood cell count; HB: hemoglobin; WBC: white blood cell count; PLT: platelet count; Tbil: total bilirubin; ALT: alanine aminotransferase; AST: aspartate aminotransferase; eGFR: estimate glomerular filtration rate; CK: creatine kinase; LDH: lactate dehydrogenase; FIB: fibrinogen; AGLU: admission blood glucose; GGT: γ-glutamyltransferase; LDL-C: low-density lipoprotein cholesterol TG: triglyceride; HDL-C: high-density lipoprotein cholesterol; NT-proBNP: N-terminal pro-B-type natriuretic peptide; cTnT: troponin T;

**Supplementary Table 2**: Detailed covariate lists for Cox model.

| Covariate block | Variables included |
| --- | --- |
| Demographics | sex, age |
| Comorbidities | DM, HT, HF, COPD, CAD, Stroke/TIA, SE, Malignant tumor |
| Medications | OAC, antiplatelet, β-blocker, statin, ARNI/ACEI/ARB, SGLT2 |
| Labs | eGFR, AGLU, LDH, FIB, ALT, AST, CK, WBC, PLT, HB |
| Advanced biomarkers | GGT, LDL-C, TG, HDL-C, NT-proBNP, cTnT |

DM: diabetes mellitus; HT: hypertension; HF: heart failure; COPD: chronic obstructive pulmonary disease; CAD: coronary artery disease; TIA: transient ischemic attack; SE: systemic embolism; OAC: oral anticoagulant; ARNI: angiotensin receptor neprilysin inhibitor; ACEI: angiotensin converting enzyme inhibitors; ARB: angiotensin receptor blocker; SGLT2: sodium-dependent glucose transporters 2; eGFR: estimate glomerular filtration rate; AGLU: admission blood glucose; LDH: lactate dehydrogenase; FIB: fibrinogen; ALT: alanine aminotransferase; AST: aspartate aminotransferase; CK: creatine kinase; WBC: white blood cell count; PLT: platelet count; HB: hemoglobin; GGT: γ-glutamyltransferase; LDL-C: low-density lipoprotein cholesterol TG: triglyceride; HDL-C: high-density lipoprotein cholesterol; NT-proBNP: N-terminal pro-B-type natriuretic peptide; cTnT: troponin T;

**Supplementary Table 3**: Baseline characteristics for external validation cohort.

|  | **T1(n=226)** | **T2(n=164)** | **T3(n=221)** | **T4(n=755)** | ***P*.value** |
| --- | --- | --- | --- | --- | --- |
|  | **UAR<6.890** | **UAR:6.890-8.728** | **UAR:8.728-11.142** | **UAR>11.142** |  |
| Male | 118 (52.2%) | 94 (57.3%) | 131 (59.3%) | 480 (63.6%) | 0.016 |
| Age | 70.0 [61.0;78.0] | 71.0 [64.0;77.0] | 73.0 [64.0;81.0] | 75.0 [65.0;82.0] | <0.001 |
| Weight (kg) | 73.3 [61.6;87.8] | 75.9 [64.5;92.4] | 76.1 [64.9;90.8] | 79.8 [67.6;95.7] | 0.002 |
| Height (m) | 1.68 [1.62;1.78] | 1.67 [1.60;1.77] | 1.68 [1.62;1.76] | 1.70 [1.63;1.78] | 0.075 |
| CHA2DS2-VASc | 3.00 [2.00;4.00] | 3.00 [2.00;4.00] | 3.00 [2.00;4.00] | 3.00 [2.00;5.00] | <0.001 |
| **Comorbidity (%)** |  |  |  |  |  |
| DM | 51 (22.6%) | 36 (22.0%) | 47 (21.3%) | 238 (31.5%) | 0.001 |
| HT | 142 (62.8%) | 108 (65.9%) | 140 (63.3%) | 501 (66.4%) | 0.713 |
| HF | 68 (30.1%) | 53 (32.3%) | 85 (38.5%) | 391 (51.8%) | <0.001 |
| Stroke/TIA | 24 (10.6%) | 11 (6.71%) | 12 (5.43%) | 43 (5.70%) | 0.059 |
| SE | 5 (2.21%) | 0 (0.00%) | 3 (1.36%) | 14 (1.85%) | 0.273 |
| CAD | 38 (16.8%) | 23 (14.0%) | 31 (14.0%) | 147 (19.5%) | 0.150 |
| PAD | 0 (0.00%) | 0 (0.00%) | 0 (0.00%) | 1 (0.13%) | 1.000 |
| COPD | 16 (7.08%) | 19 (11.6%) | 27 (12.2%) | 128 (17.0%) | 0.001 |
| OSAS | 9 (3.98%) | 12 (7.32%) | 14 (6.33%) | 44 (5.83%) | 0.536 |
| Hyperthyroidism | 2 (0.88%) | 0 (0.00%) | 2 (0.90%) | 8 (1.06%) | 0.711 |
| Hypothyroidism | 30 (13.3%) | 23 (14.0%) | 26 (11.8%) | 116 (15.4%) | 0.559 |
| Malignant tumor | 120 (53.1%) | 88 (53.7%) | 125 (56.6%) | 284 (37.6%) | <0.001 |
| **Laboratory tests** |  |  |  |  |  |
| RBC (k/ul) | 3.55 [3.01;4.03] | 3.44 [2.85;4.01] | 3.40 [2.90;3.98] | 3.47 [2.95;4.01] | 0.628 |
| HB (g/dl) | 11.0 [9.20;12.4] | 10.6 [8.90;12.1] | 10.5 [9.00;12.0] | 10.5 [8.90;12.1] | 0.154 |
| WBC (k/ul) | 8.00 [4.70;13.8] | 8.50 [5.68;12.8] | 8.40 [5.70;14.5] | 10.1 [6.90;15.9] | <0.001 |
| PLT (k/ul) | 188 [117;260] | 173 [93.0;258] | 171 [103;238] | 188 [122;269] | 0.069 |
| Tbil(mg/dl) | 0.60 [0.40;1.00] | 0.60 [0.40;1.08] | 0.60 [0.40;1.10] | 0.70 [0.40;1.30] | 0.004 |
| ALT (IU/L) | 24.0 [16.0;38.2] | 24.0 [15.0;41.0] | 22.0 [14.0;41.2] | 24.0 [14.0;52.0] | 0.801 |
| AST (IU/L) | 28.5 [20.0;46.0] | 29.5 [19.0;52.8] | 28.0 [18.2;55.0] | 34.0 [20.0;70.0] | 0.004 |
| eGFR  (mL/min/1.73 m²) | 86.0 [57.2;96.7] | 78.1 [50.8;92.8] | 65.7 [45.1;87.3] | 41.2 [22.3;64.0] | <0.001 |
| CK (IU/L) | 66.0 [30.2;186] | 67.0 [30.0;162] | 58.0 [29.0;181] | 83.0 [38.5;224] | 0.066 |
| LDH (IU/L) | 279 [205;395] | 278 [208;485] | 275 [208;394] | 309 [220;509] | 0.002 |
| FIB (mg/dl) | 389 [262;551] | 357 [272;499] | 376 [251;507] | 357 [231;525] | 0.496 |
| AGLU (mg/dl) | 124 [101;162] | 114 [93.0;143] | 114 [97.0;149] | 118 [98.0;155] | 0.061 |
| **Medication use (%)** |  |  |  |  |  |
| OAC | 69 (30.5%) | 66 (40.2%) | 68 (30.8%) | 312 (41.3%) | 0.003 |
| Antiplatelet drugs | 86 (38.1%) | 63 (38.4%) | 81 (36.7%) | 361 (47.8%) | 0.002 |
| ANRI/ACEI/ARB | 53 (23.5%) | 42 (25.6%) | 57 (25.8%) | 216 (28.6%) | 0.430 |
| β blocker: | 186 (82.3%) | 137 (83.5%) | 179 (81.0%) | 609 (80.7%) | 0.824 |
| Statin: | 86 (38.1%) | 62 (37.8%) | 79 (35.7%) | 323 (42.8%) | 0.192 |
| SGLT2: | 2 (0.88%) | 1 (0.61%) | 0 (0.00%) | 3 (0.40%) | 0.447 |

UAR: uric acid-to-albumin ratio; DM: diabetes mellitus; HT: hypertension; HF: heart failure; TIA: transient ischemic attack; SE: systemic embolism; CAD: coronary artery disease; PAD: peripheral arterial disease; COPD: chronic obstructive pulmonary disease; OSAS: obstructive sleep apnea syndrome; RBC: red blood cell count; HB: hemoglobin; WBC: white blood cell count; PLT: platelet count; Tbil: total bilirubin; ALT: alanine aminotransferase; AST: aspartate aminotransferase; eGFR: estimate glomerular filtration rate; CK: creatine kinase; LDH: lactate dehydrogenase; FIB: fibrinogen; AGLU: admission blood glucose; OAC: oral anticoagulant; ARNI: angiotensin receptor neprilysin inhibitor; ACEI: angiotensin converting enzyme inhibitors; ARB: angiotensin receptor blocker; SGLT2: sodium-dependent glucose transporters 2

**Supplementary Table 4**: Full multivariable Cox regression results for primary cohort.

|  | **HR** | **95%CI** | ***P*.value** |
| --- | --- | --- | --- |
| UAR (per 1-SD) | 1.130 | 1.006-1.269 | 0.039 |
| Sex (male =0) | 0.828 | 0.636-1.078 | 0.160 |
| Age | 0.992 | 0.966-1.018 | 0.545 |
| tt(Age) | 1.010 | 1.003-1.016 | 0.005 |
| BMI | 0.959 | 0.920-0.997 | 0.036 |
| DM | 1.034 | 0.757-1.414 | 0.832 |
| HT | 0.840 | 0.646-1.092 | 0.193 |
| HF | 1.507 | 1.160-1.958 | 0.002 |
| SE | 1.742 | 0.992-3.059 | 0.053 |
| CAD | 0.934 | 0.705-1.239 | 0.636 |
| COPD | 1.436 | 1.056-1.953 | 0.021 |
| HB | 1.427 | 1.027-1.981 | 0.342 |
| WBC | 1.036 | 1.013-1.058 | 0.013 |
| PLT | 1.001 | 0.999-1.002 | 0.452 |
| ALT | 1.000 | 0.999-1.002 | 0.658 |
| AST | 0.999 | 0.999-1.000 | 0.124 |
| GGT | 2.092 | 1.536-2.850 | <0.001 |
| eGFR | 0.999 | 0.993-1.006 | 0.839 |
| CK | 1.964 | 1.346-2.866 | <0.001 |
| LDH | 1.001 | 1.000-1.001 | <0.001 |
| FIB | 1.000 | 0.999-1.001 | 0.762 |
| AGLU | 1.002 | 1.000-1.004 | 0.020 |
| LDL-C | 0.997 | 0.993-1.001 | 0.166 |
| TG | 0.998 | 0.996-1.000 | 0.122 |
| HDL-C | 0.989 | 0.979-0.999 | 0.027 |
| NT-proBNP | 1.000 | 1.000-1.000 | 0.022 |
| cTnT | 0.861 | 0.747-0.992 | 0.038 |
| Antiplatelet drugs | 1.048 | 0.745-1.474 | 0.787 |
| ANRI/ACEI/ARB | 0.851 | 0.597-1.215 | 0.374 |
| β blocker: | 1.134 | 0.856-1.502 | 0.379 |
| Statin: | 0.916 | 0.681-1.230 | 0.557 |
| SGLT2: | 1.427 | 0.427-4.762 | 0.562 |

HR: hazard ratio; CI: confidence interval; UAR: uric acid-to-albumin ratio; DM: diabetes mellitus; HT: hypertension; HF: heart failure; SE: systemic embolism; CAD: coronary artery disease; COPD: chronic obstructive pulmonary disease; HB: hemoglobin; WBC: white blood cell count; PLT: platelet count; ALT: alanine aminotransferase; AST: aspartate aminotransferase; GGT: γ-glutamyltransferase; eGFR: estimate glomerular filtration rate; CK: creatine kinase; LDH: lactate dehydrogenase; FIB: fibrinogen; AGLU: admission blood glucose; LDL-C: low-density lipoprotein cholesterol TG: triglyceride; HDL-C: high-density lipoprotein cholesterol; NT-proBNP: N-terminal pro-B-type natriuretic peptide; cTnT: troponin T; ARNI: angiotensin receptor neprilysin inhibitor; ACEI: angiotensin converting enzyme inhibitors; ARB: angiotensin receptor blocker; SGLT2: sodium-dependent glucose transporters 2


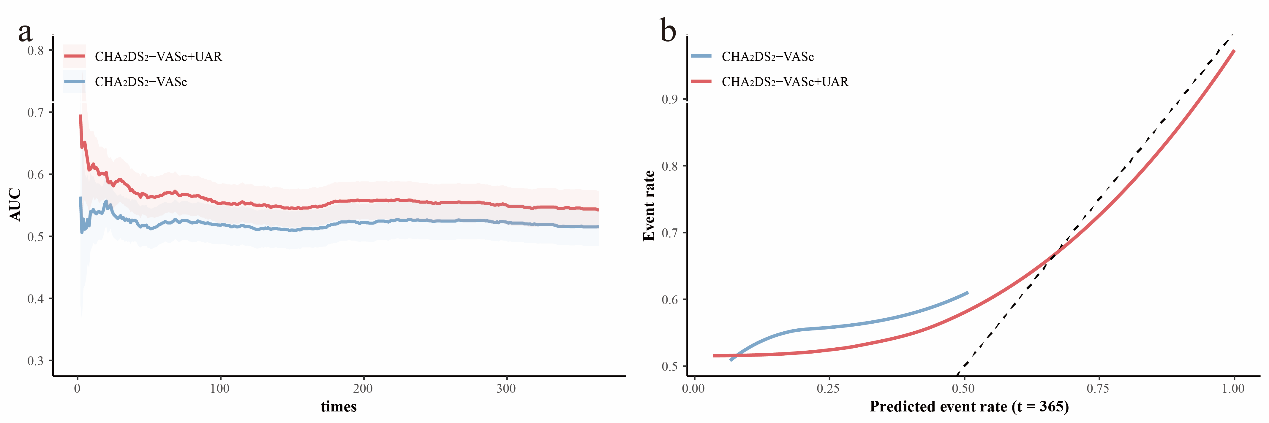
 **Supplementary Figure 1**: a: Time-dependent AUC comparison of CHA₂DS₂-VASc with and without UAR in external validation cohort. b: Calibration curves comparing CHA₂DS₂-VASc models with and without UAR in external validation cohort. AUC: area under curve; UAR: uric acid-to-albumin ratio


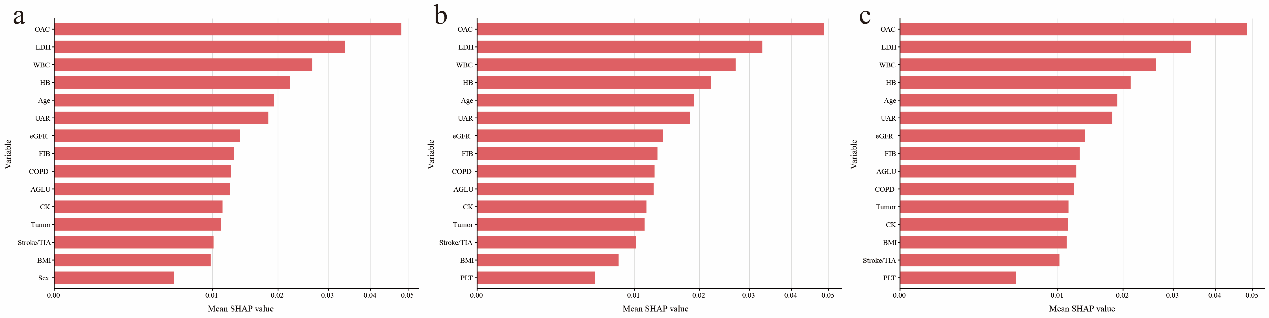


**Supplementary Figure 2**: a: RSF-SHAP variable importance in primary cohort (imputed data 2). b: RSF-SHAP variable importance in primary cohort (imputed data 3). a: RSF-SHAP variable importance in primary cohort (imputed data 4).RSF: random survival forests; SHAP: Shapley additive explanations; OAC: oral anticoagulant; LDH: lactate dehydrogenase; WBC: white blood cell count; HB: hemoglobin; UAR: uric acid-to-albumin ratio; eGFR: estimate glomerular filtration rate; FIB: fibrinogen; BMI: body mass index; AGLU: admission blood glucose; COPD: chronic obstructive pulmonary disease; CK: creatine kinase; TIA: transient ischemic attack; BMI: body mass index; PLT: platelet count


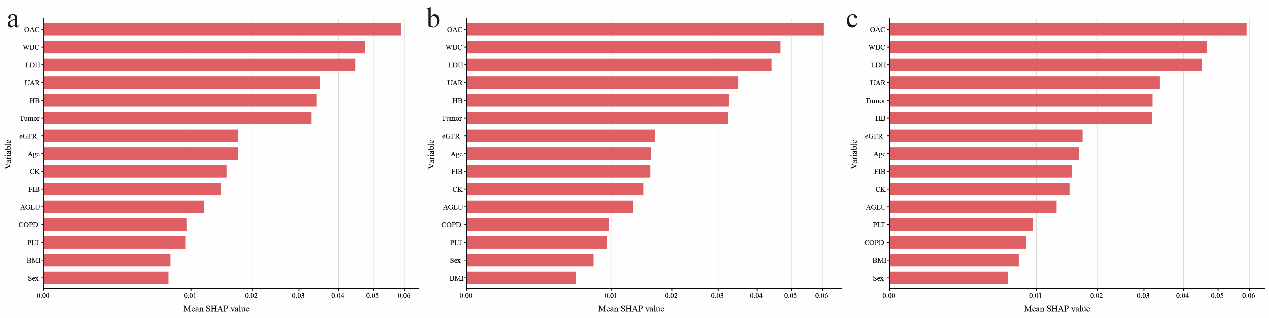


**Supplementary Figure 3**: a: RSF-SHAP variable importance in external validation cohort (imputed data 2). b: RSF-SHAP variable importance in external validation cohort (imputed data 3). a: RSF-SHAP variable importance in external validation cohort (imputed data 4).RSF: random survival forests; SHAP: Shapley additive explanations; OAC: oral anticoagulant; LDH: lactate dehydrogenase; WBC: white blood cell count; HB: hemoglobin; UAR: uric acid-to-albumin ratio; eGFR: estimate glomerular filtration rate; FIB: fibrinogen; BMI: body mass index; AGLU: admission blood glucose; COPD: chronic obstructive pulmonary disease; CK: creatine kinase; TIA: transient ischemic attack; BMI: body mass index; PLT: platelet count


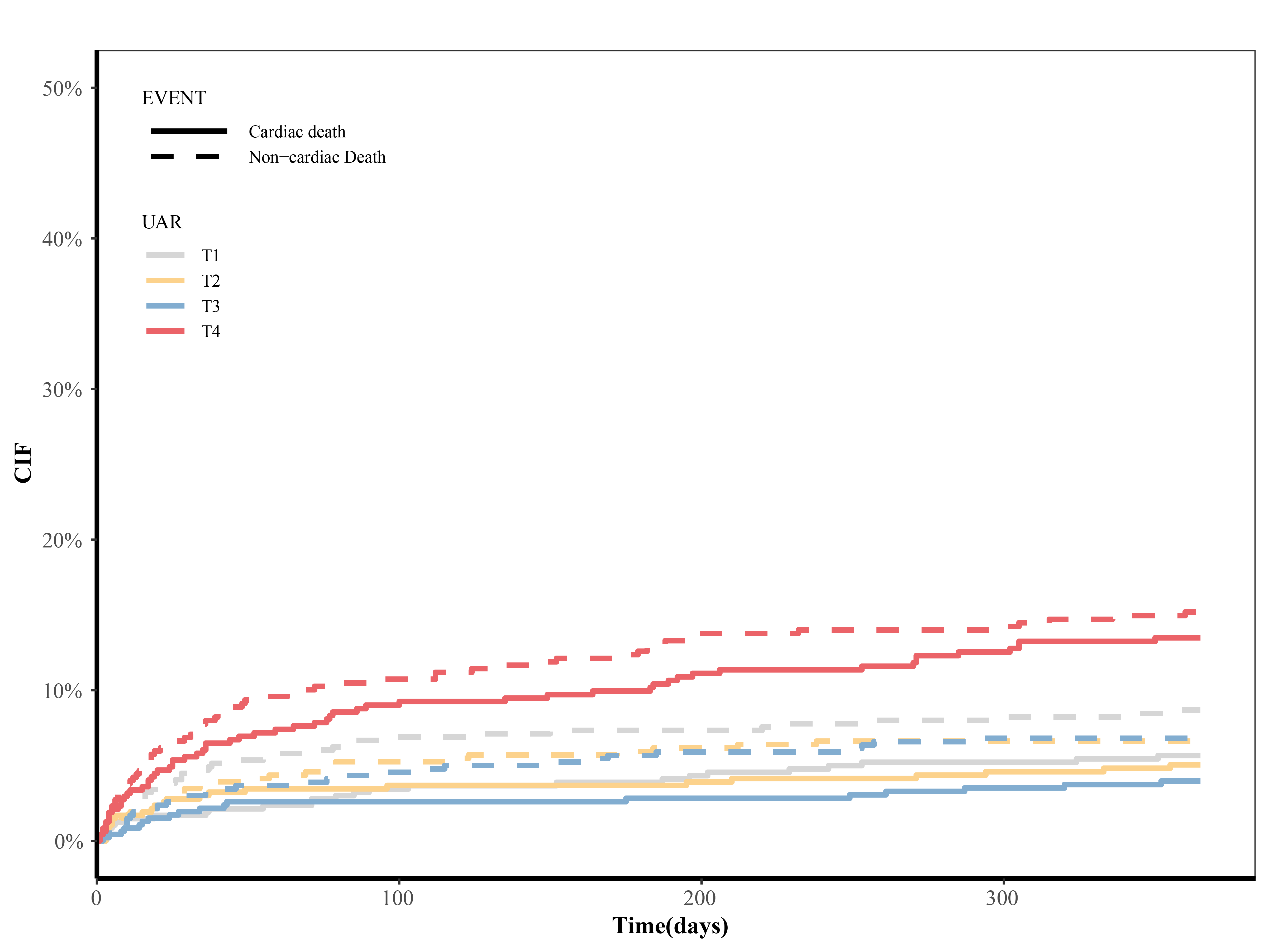


**Supplementary Figure 4**: Cumulative incidence of 1-year cardiovascular mortality by UAR quartiles with non-cardiovascular death as a competing risk. CIF: Cumulative incidence function; UAR: uric acid-to-albumin ratio
