# Supplementary material for: Uric acid-to-albumin ratio as a cardiometabolic marker for predicting adverse outcomes in patients with atrial fibrillation: evidence from two independent cohorts
Source: Front Endocrinol (Lausanne). 2026 Feb 13;17:1786997. doi: 10.3389/fendo.2026.1786997 (PMC12945761; doi:10.3389/fendo.2026.1786997)
Supplement: Supplementary file 2 [file DataSheet2.docx]

Supplementary Material 2

# Supplemental Method

Baseline characteristics were summarized across UAR quartiles to describe patient profiles. Continuous variables were reported as median (IQR) and compared using the Kruskal–Wallis test. Categorical variables were reported as n (%) and compared using the chi-square test or Fisher’s exact test.

Missing values in covariates were addressed with multiple imputation by chained equations, performed separately in the West China Hospital cohort and the MIMIC cohort. Twenty imputed datasets were created for each cohort. Results from imputed datasets were combined using Rubin’s rules.

In the primary cohort, Kaplan–Meier curves were constructed to visualize 1-year all-cause mortality across UAR quartiles and compared using the log-rank test. UAR was further modeled as a continuous variable using restricted cubic splines (RCS) to assess potential nonlinear associations, with the number and placement of knots selected based on the Akaike information criterion. Cox proportional hazards models were used to estimate hazard ratios (HRs) and 95% confidence intervals (CIs) for the association between UAR and mortality. UAR was modeled as a continuous variable per one standard deviation (SD) increase. A sequence of models with increasing degrees of adjustment was fitted to evaluate the robustness of the association across clinically relevant variables: Model 1 included UAR alone; Model 2 additionally adjusted for age and sex; Model 3 further included BMI, comorbidities and medication use; Model 4 additionally adjusted for available laboratory variables. Proportional hazards assumptions were evaluated using Schoenfeld residuals. For age, non-proportionality was addressed by allowing a time-varying effect, while other violations were handled by stratification when appropriate. Creatine kinase (CK), GGT, and HB were modeled as categorical variables using clinically defined cut points. External validation was performed in the MIMIC cohort with UAR scaled using the SD from the primary cohort.

To quantify the additional prognostic value of UAR beyond established clinical risk assessment, we compared a Cox model including CHA₂DS₂-VASc score alone with a model that additionally included UAR. Discrimination was assessed using Harrell’s C-index and time-dependent AUC. Calibration was evaluated with calibration plots, and clinical utility was examined using decision curve analysis. For external validation, the primary model was applied to the validation set, resulting in risk prediction. Discrimination was assessed using the C-index and time-dependent AUC, and calibration plots was also conducted. Furthermore, the improvement after adding UAR was quantified using continuous time-to-event net reclassification improvement (NRI) and integrated discrimination improvement (IDI) for two cohorts.

Exploratory machine learning analyses were conducted to assess potential nonlinear relationships and to evaluate the relative importance of UAR compared with other clinical variables. Following the principle of at least 10 outcome events per predictor, 29 clinically relevant variables, including UAR, were selected from one imputed primary dataset. Random survival forests (RSF), extreme gradient boosting (XGBoost), and gradient boosting machine (GBM) models were trained using 5-fold cross-validation. The best-performing model structure was subsequently applied to all imputed datasets in both cohorts, and performance metrics were averaged across imputations. Model interpretability focused on variable importance and directionality using Shapley additive explanations (SHAP), and the robustness of findings was examined by repeating tuning and SHAP analyses in additional 3 imputed datasets.

Prespecified subgroup analyses in the primary cohort were carried out by age (<65 vs ≥65 years), sex, eGFR (>60 vs ≤60 mL/min/1.73 m²), BMI (>28 vs ≤28 kg/m²), and the presence of HF, DM, HT, stroke/TIA, malignant tumor, statin use and OAC use. As sensitivity analyses, we additionally fitted Cox proportional hazards models with further adjustment for γ-glutamyltransferase, lipid parameters (LDL-C, HDL-C, TG) and cardiac biomarkers (NT-proBNP and cTnT) in the primary cohort only. For 1-year cardiovascular mortality, we conducted Cox proportional hazards models with and without adjustment for the CHA₂DS₂-VASc score. Then, we treated non-cardiovascular death as the competing event, and estimated cumulative incidence functions and fitted Fine–Gray subdistribution hazard models. Moreover, we examined the associations of UAR with 4-year cardiovascular and all-cause mortality using Cox models and RCS.

All analyses were performed in R (version 4.5.1). A two-sided P value <0.05 was considered statistically significant.
